# Light People: Professor Che Ting Chan, curiosity drives to create the impossibilities

**DOI:** 10.1038/s41377-024-01497-z

**Published:** 2024-06-21

**Authors:** Tingting Sun, Ying Xiong

**Affiliations:** 1 Light Publishing Group, Changchun Institute of Optics, Fine Mechanics and Physics, Chinese Academy of Sciences, No. 3888 Dong Nanhu Road, Changchun, 130033 China; 2 College of Intelligence Science and Technology, National University of Defense Technology, No. 109 Deya Road, Changsha, 410073 China

**Keywords:** Metamaterials

## Abstract

“When something is said to be impossible, there are two points for researchers to initially clarify: whether it really is forbidden by the laws of nature; or whether it is simply that no material that currently exists in nature can do that.” Metamaterials are such magical beings, which have physical properties like invisibility, negative refraction, super-resolution, and perfect absorption that are absent from natural materials. It has been rated by Science as one of the top ten scientific and technological breakthroughs affecting human beings in the 21st century.

In this issue of Light People, we spoke with a “magic” creator, Professor Che Ting Chan, the Associate Vice-President (Research & Development) of the Hong Kong University of Science and Technology (HKUST), Member of the Hong Kong Academy of Sciences and Fellow of the American Physical Society. He has researched a number of theoretical problems in material physics, investigated the theory behind what they seek to achieve, and modulated light (electromagnetism) and acoustic waves through metamaterials. In the following, let’s take a closer look at Professor Che Ting Chan’s research life, and appreciate his style and the background of his accomplishment.

## Abstract (web summary)

Prof. Che Ting Chan has researched theoretical problems in material physics, investigated the theory behind what they seek to achieve, and modulated light (electromagnetism) and acoustic waves through metamaterials.

**Short Bio**: Prof. Che Ting Chan serves as the Associate Vice-President (Research & Development) of the Hong Kong University of Science and Technology (HKUST), the Daniel C K Yu Professor of Science, Chair Professor in the Department of Physics, and the Director of the Research Office of HKUST. He is also a Member of the Hong Kong Academy of Sciences and Fellow of the American Physical Society. Prof. Chan received his BSc degree from the University of Hong Kong in 1980 and his Ph.D. degree from the University of California at Berkeley in 1985. Then he was employed at Ames Laboratory-USDOE and joined HKUST in 1995. He was elected as a Fellow of the American Physical Society in 1996 and a Member of the Hong Kong Academy of Sciences in 2021. He was awarded significant international honors including the Brillouin Medal and the Achievement in Asia Award. Prof. Chan has published over 150 papers in prestigious international journals including Nature, Science, Nature Photonics, Light: Science & Applications, etc. with a total of more than 60,000 citations. His primary areas of interest include surface physics, nanomaterials, metamaterials, and light manipulation. He is now the Associate EIC of ACS Photonics and has previously served on the editorial boards of Applied Physics, Physical Review B, etc.

**Figure:**
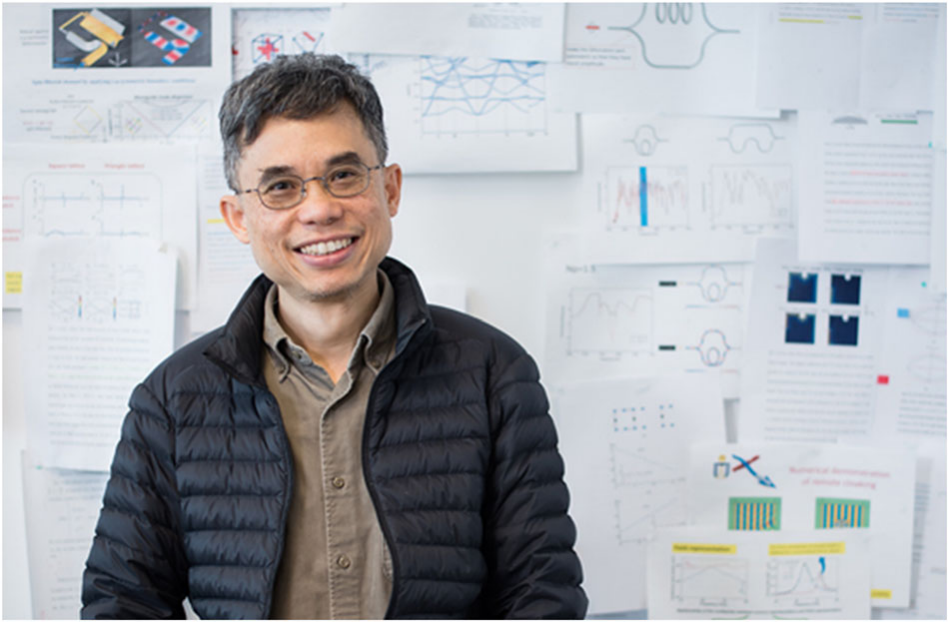

**Q1: The novel topological phenomena in the field of photonic crystals and metamaterials have been attracting much attention, your proposal for the in-plane nodal chain that widely exist in photonic systems, with a complete presentation and experimental validation of the nodal chain structure** ^1^ **. Since its publication in Light: Science & Applications (Light), this paper has drawn significant interest from experts and scholars worldwide. Could you kindly discuss the innovation of this work and its potential applications?**

A1: Degeneracies play a crucial role in topological physics due to their unique properties and significance in characterizing topological materials. Degeneracy in band structure is intimately connected to Berry phases, which are central concepts in topological physics. Nodal lines are particular forms of degeneracy structures. Their geometrical structure in momentum space is interesting and are associated with the geometric properties of energy bands and play a crucial role in characterizing topological materials. This study also shows that the zero-frequency, zero-momentum point in the band structure is actually highly non-trivial and is the source of topological structures in some photonic systems.

At present, this research enhances our comprehension of nodal structures in momentum space. This understanding holds the potential to unlock new avenues for developing topological photonic devices, ultimately leading to the creation of robust energy and signal transportation methods that are impervious to disorder or imperfections.

**Q2: You have been dedicated to the research on photonic crystals, could you outline the key challenges of this field? How do you see the future development of photonic crystals?**

A2: One of the major challenges in photonic crystals is developing reliable and cost-effective fabrication techniques that can achieve the desired structures with high precision and control. The fabrication process needs to overcome challenges such as scalability, reproducibility, and integration with other technologies. Photonic crystals can suffer from losses that limit their performance. Reducing optical losses is essential. Future: Further exploration and optimization of photonic crystal structures can significantly enhance light-matter interaction, enabling novel applications in areas such as sensing, quantum information processing, and nonlinear optics. Integration is important. Hopefully, the continued progress in fabrication techniques and device integration will enable the seamless integration of photonic crystal devices into integrated photonics platforms. This integration will lead to compact, multifunctional, and highly efficient photonic systems for communication, computing, and sensing.

**Figure:**
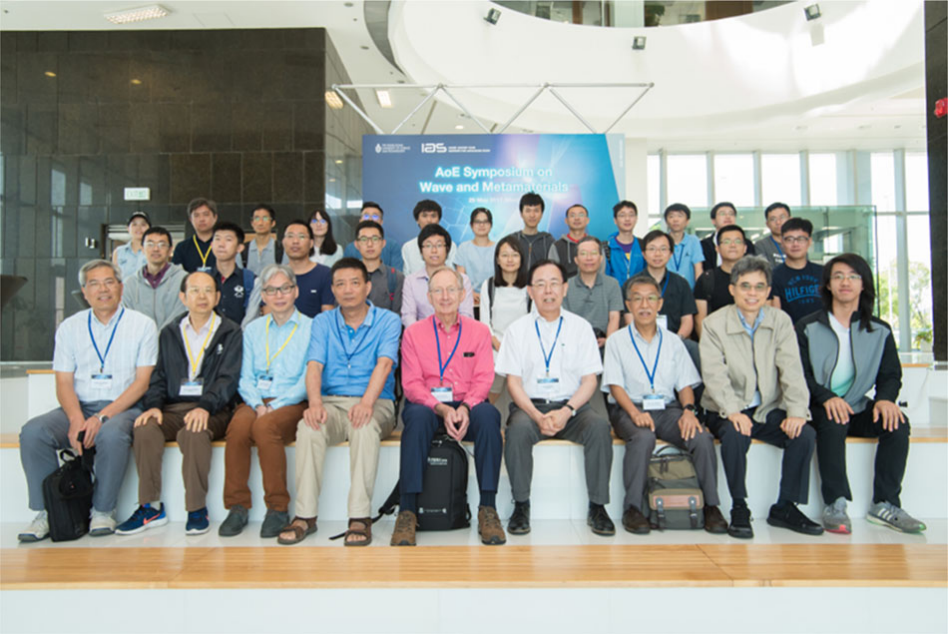
AoE Symposium on Wave and Metamaterials organized by Prof. Chan

**Q3: In 2023, you published a work in Light on the expansion of chiral zeroth Landau levels from the three-dimensional to the two-dimensional regime** ^2^ **, could you briefly introduce this research work?**

A3: We have demonstrated the generation of a synthetic in-plane magnetic field by introducing an inhomogeneous effective mass through the breaking of local parity-inversion symmetries. This enabled the induction of zeroth-order chiral Landau levels and the experimental observation of their one-way propagation characteristics. Furthermore, we have experimentally tested the robust transport of the chiral zeroth mode against defects in the system. Our findings offer a new approach to realizing chiral Landau levels using pseudo-magnetic fields.

The primary focus of this paper is on the realization of synthetic gauge fields. Synthetic gauge fields have emerged as a powerful tool because they can emulate the effects of real magnetic fields. Unlike real magnetic fields, which typically require the presence of charged particles or currents, synthetic gauge fields are created through tailored photonic structures and interactions. One of the significant advantages of synthetic gauge fields is their enhanced controllability and tunability. This flexibility empowers us to create novel functionalities and tailor properties that are not easily achievable with real magnetic fields.

**Q4: You detected experimentally non-Abelian topological charges through experimentation for the first time, and verified the bulk-edge correspondence relating to non-Abelian topological charges** ^3^ **.This pioneering work, published in Nature, offered constructive guidance for subsequent research in non-Abelian topology field. Recently, your review article on this field published in Science has sparked widespread attention and discussion** ^4^ **. What do you think will be this field’s next big breakthrough? Could you provide a brief overview of the latest developments in this field by your team?**

A4: Conventional topological materials are commonly described by integers such as winding numbers and Chern numbers. However, non-Abelian topological systems introduce a different perspective by employing non-integer entities like quaternions, which resemble matrices. Unlike the traditional approach of describing the topology of one band at a time, these entities collectively characterize a group of bands. This novel viewpoint offers a fresh understanding that goes beyond conventional studies. Currently, we are expanding this concept from Hermitian systems to non-Hermitian systems. This extension allows us to explore the unique properties and behaviors arising from non-Hermitian interactions. By delving into the realm of non-Hermitian topological systems, we aim to uncover new insights and unveil intriguing phenomena that were previously unexplored. This research direction may open up exciting possibilities for advancing our understanding of topological physics in non-Hermitian contexts.

**Figure:**
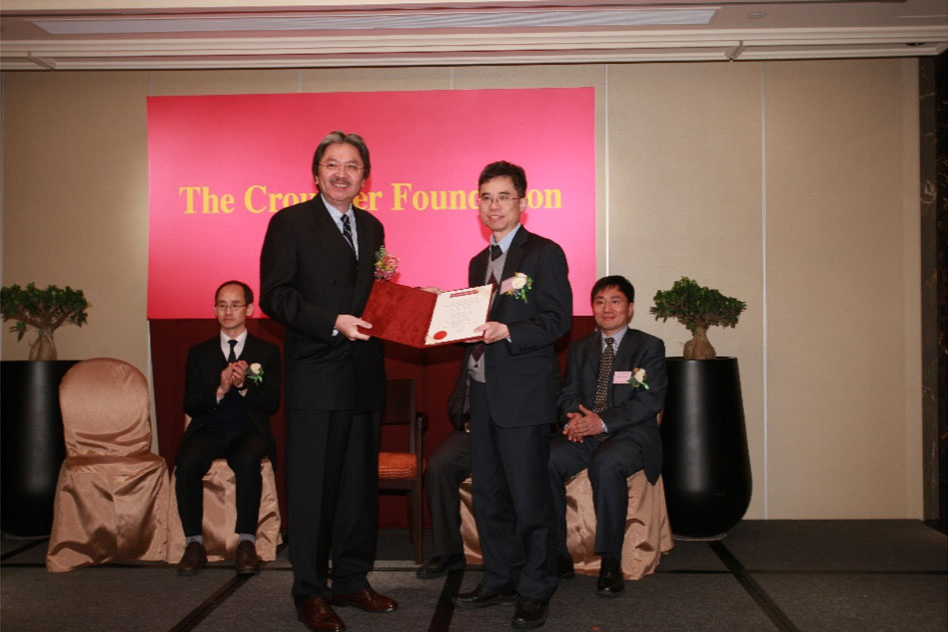
Prof. Chan was awarded the Croucher Fellowship

**Q5: In addition to being an expert of the electromagnetic metamaterials field, you have made significant contributions to the field of acoustic topology. The most impressive work is the one published in Science on locally resonant sonic materials** ^5^ **, which has been cited 5485 times at present and had a significant influence to the corresponding fields. How did you achieve this major innovation? What about the follow-up?**

A5: Indeed, this paper is sometimes recognized as a pioneering contribution to the field of acoustic metamaterials. Although my personal involvement is focused on theoretical aspects rather than participation in knowledge transfer, I am aware that some colleagues involved in this project have subsequently transferred the knowledge gained. Notably, they have made significant strides in producing practical meta-acoustic products which can trace their origin to the ideas to this paper.

This research stems from an idea conceived by Prof. Ping Sheng from HKUST, who posited that while static negative mass may not be feasible, there exists the possibility of achieving negative effective mass at a finite frequency, at least in theory. My contribution to this endeavor involved developing the theoretical framework and conducting computations. However, the true significance of this work also lies in its experimental realization, which was accomplished by Prof. Sheng’s experimental team and other experimentalist collaborators. To achieve this great work, we all paid a lot of efforts, thanks to my collaborators.

**Q6: Your papers have got over 60,000 citations in total, with eight papers each cited more than 1000 times, and your H-index is an impressive 112, a level that many researchers can only aspire to. What guidelines or suggestions do you have for cultivating the innovation ability and scientific research level of the group?**

A6: We need to promote a culture of excellence and rigor in research. Knowledge-driven research is not just about publishing papers. It is more about finding answers to questions that do not have an answer before our work. To facilitate that, we want to encourage open communication, collaboration, and interactions within the research group. Promote an inclusive and supportive atmosphere where ideas can be freely shared, discussed, and refined. Collaboration often leads to fresh perspectives and breakthrough discoveries.

**Figure:**
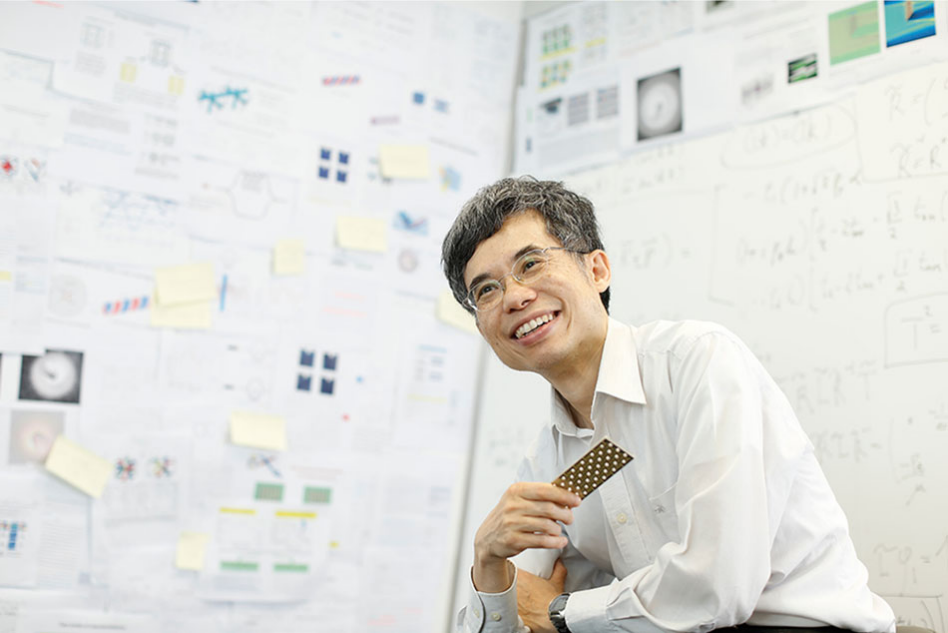
Prof. Chan sharing his passion for research with young scholars

**Q7: After receiving your bachelor’s degree from the University of Hong Kong, you went to the University of California, Berkeley, USA to pursue a doctor degree in Physics. How did this experience influence your later research career?**

A7: The Berkeley experience has been truly transformative and eye-opening for me. Having spent my undergraduate years at HKU, which was a teaching university at that time, I have not been exposed to research at an international level. However, my time at Berkeley provided me with a profound insight into world-class research practices. It’s fair to say that in graduate school, we can acquire a solid foundation in physics through self-study. Nevertheless, it is the combination of a supportive environment and a knowledgeable Ph.D. advisor that truly illuminates the path towards conducting meaningful research.

**Q8: What advises do you have for the researchers when they encounter difficulties and challenges in their work?**

A8: In the field of physics, the supply of qualified individuals far exceeds the demand for stable job positions. Even after obtaining a job, there is immense pressure to publish research quickly. This fast-paced environment can be quite overwhelming. It would be truly remarkable if scientists could be afforded the luxury of time and freedom to delve into complex and meaningful problems that require deep contemplation. Unfortunately, such an environment seems increasingly elusive in today’s world. To address these challenges, it is crucial to cultivate adaptability and resilience.

**Figure:**
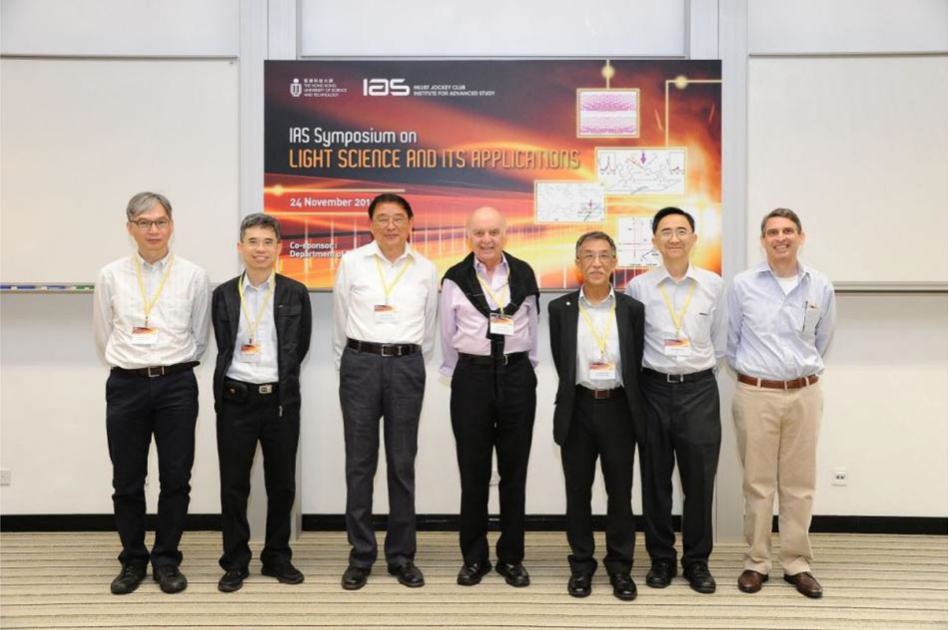
IAS Symposium on Light Science and Its Applications organized by Prof. Chan

**Q9: We heard that you’d been looking for the answers to all the questions about natural phenomena that captivated you as a child. This curiosity has reportedly led to important scientific breakthroughs, like the creation of “Harry Potter’s Invisibility Cloak,” which used metamaterials to manipulate light and waves to make objects invisible. You often share your passion to science with your students and discuss the science in movies such as Harry Potter and Star Trek. Would you like to share some of your fantasies with us?**

A9: We often indulge in fantasies that seem achievable only in movies but appear far-fetched in reality. However, with continuous advancements in science and technology, it becomes fascinating to explore the realm of possibilities without violating upon the laws of nature. It is precisely this exploration that serves as a catalyst, demonstrating that what may seem impossible at first glance can, in fact, be accomplished. The ever-evolving landscape of scientific knowledge facilitates the unveiling of new frontiers and instills a sense of wonder, showing the potential for turning the seemingly impossible into a reality.

**Q10: In addition to your accomplishments in research, you have been recognized for your teaching abilities with awards from HKUST, including as the Common Core Course Excellence Award and the Michael Gale Medal for Distinguished Teaching. What parallels do you think exist between research and teaching? Would you like to share your great experience?**

A10: I haven’t undergone formal “teacher training,” which means my expertise in teaching methods and pedagogical technology may not be as advanced. However, I have identified two common challenges in teaching. Firstly, certain subjects can be inherently dry and complex, making it crucial to find creative ways to engage students and make the material more accessible and interesting. Secondly, classrooms often consist of students with varying levels of preparation and understanding. To address this diversity, it is important to carefully consider the needs of each student while preparing course materials, ensuring that the content accommodates different learning styles and provides support to those who may require additional assistance. By acknowledging these challenges and actively working towards overcoming them, we can create a more inclusive and enriching learning environment for all students.

**Figure:**
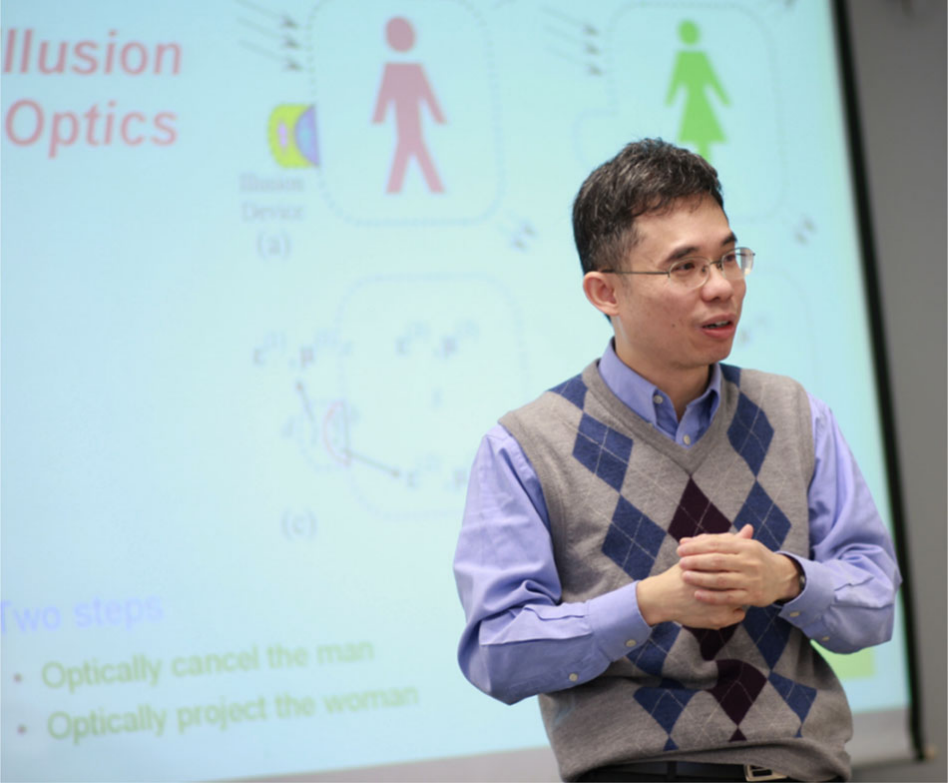
Prof. Chan explains illusion optics

**Q11: How do you stay inspired and enthusiastic when faced with a variety of responsibilities like managing a university, leading research, and education teaching? Outside of work, what interests do you have to balance your work and life?**

A11: To be honest. During my younger years, I dedicated most of my time to work, neglecting the importance of achieving a healthy work-life balance. I certainly can’t be considered a role model in this regard. I do some reading outside work. In my younger days, I read a diverse range of literature, predominantly Chinese works spanning from martial arts novels to classical Chinese literature. In recent years, my reading preferences have shifted towards delving into the realms of history.

**Q12: We really appreciate your great support of Light, particularly, you presented a wonderful keynote talk for domestic and foreign researchers at the Asia Light Conference in this March, which is co-organized by Light Publishing Group. What expectations and suggestions do you have for Light’s future development?**

A12: Congratulations on the success of your journal! It is impressive to see how quickly it has risen to become one of the highest impact factor journals in this field. I encourage you to maintain the momentum. The future of the journal certainly looks promising, and I have no doubt that it will continue to shine brightly.

**Figure:**
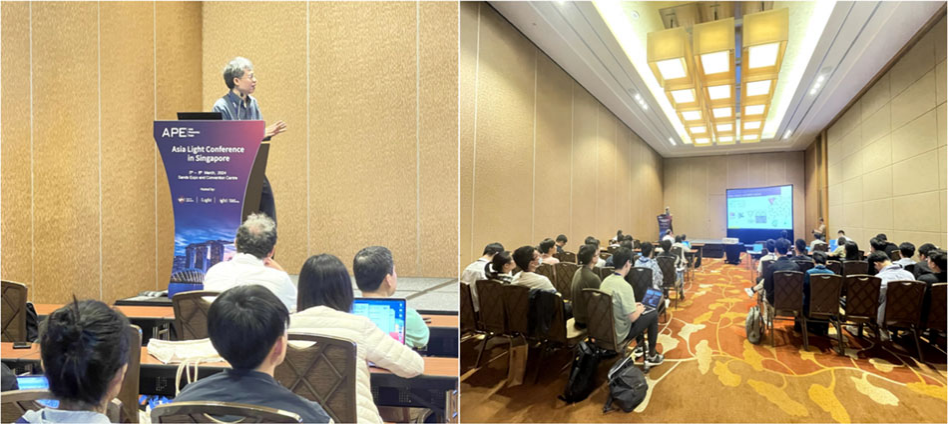
Prof. Chan giving keynote talk at Asia Light Conference in Singapore
